# 3-Bromo-*N*′-(3,5-dichloro-2-hydroxy­benzyl­idene)benzohydrazide

**DOI:** 10.1107/S1600536808041378

**Published:** 2008-12-10

**Authors:** Chuan-Gao Zhu, Yi-Jun Wei, Qi-Yong Zhu

**Affiliations:** a Department of Chemistry, Huainan Normal College, Huainan 232001, People’s Republic of China

## Abstract

The title compound, C_14_H_9_BrCl_2_N_2_O_2_, was prepared by the reaction of 3,5-dichloro-2-hydroxy­benzaldehyde and 3-bromo­benzohydrazide in methanol. The dihedral angle between the two benzene rings is 13.0 (2)°. An intra­molecular O—H⋯N hydrogen bond is observed. The mol­ecules are linked into chains along the *c* axis by inter­molecular N—H⋯O hydrogen bonds.

## Related literature

For the synthesis of Schiff bases, see: Akitsu & Einaga (2006 ▶); Butcher *et al.* (2005 ▶); Habibi *et al.* (2007 ▶); Pradeep (2005 ▶). For related structures, see: Bao & Wei (2008 ▶); Odabaşoğlu *et al.* (2007 ▶); Wang *et al.* (2006 ▶); Wei *et al.* (2008 ▶); Yathirajan *et al.* (2007 ▶); Yehye *et al.* (2008 ▶); Zhu *et al.* (2007 ▶).
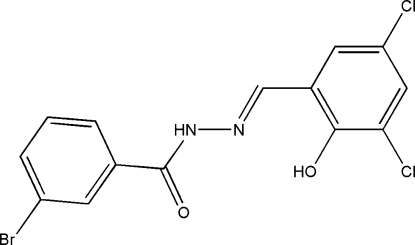


## Experimental

### 

#### Crystal data

C_14_H_9_BrCl_2_N_2_O_2_
*M* *_r_* = 388.04
Monoclinic, 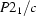

*a* = 8.272 (2) Å
*b* = 22.366 (3) Å
*c* = 8.237 (2) Å
β = 104.014 (2)°
*V* = 1478.6 (5) Å^3^
*Z* = 4
Mo *K*α radiation
μ = 3.15 mm^−1^
*T* = 298 (2) K
0.23 × 0.23 × 0.22 mm

#### Data collection

Bruker SMART 1000 CCD area-detector diffractometer
Absorption correction: multi-scan (*SADABS*; Sheldrick, 1996 ▶) *T*_min_ = 0.495, *T*_max_ = 0.513
8590 measured reflections
3224 independent reflections
2144 reflections with *I* > 2σ(*I*)
*R*_int_ = 0.035

#### Refinement

*R*[*F* ^2^ > 2σ(*F* ^2^)] = 0.036
*wR*(*F* ^2^) = 0.075
*S* = 0.98
3224 reflections
194 parameters
1 restraint
H atoms treated by a mixture of independent and constrained refinement
Δρ_max_ = 0.32 e Å^−3^
Δρ_min_ = −0.29 e Å^−3^

### 

Data collection: *SMART* (Bruker, 2002 ▶); cell refinement: *SAINT* (Bruker, 2002 ▶); data reduction: *SAINT*; program(s) used to solve structure: *SHELXS97* (Sheldrick, 2008 ▶); program(s) used to refine structure: *SHELXL97* (Sheldrick, 2008 ▶); molecular graphics: *SHELXTL* (Sheldrick, 2008 ▶); software used to prepare material for publication: *SHELXTL*.

## Supplementary Material

Crystal structure: contains datablocks global, I. DOI: 10.1107/S1600536808041378/ci2740sup1.cif

Structure factors: contains datablocks I. DOI: 10.1107/S1600536808041378/ci2740Isup2.hkl

Additional supplementary materials:  crystallographic information; 3D view; checkCIF report

## Figures and Tables

**Table 1:** Hydrogen-bond geometry (Å, °)

*D*—H⋯*A*	*D*—H	H⋯*A*	*D*⋯*A*	*D*—H⋯*A*
O1—H1⋯N1	0.82	1.88	2.598 (3)	145
N2—H2⋯O2^i^	0.89 (1)	2.03 (1)	2.898 (3)	165 (3)

Symmetry code: (i) 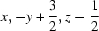.

**Fig. 1.**
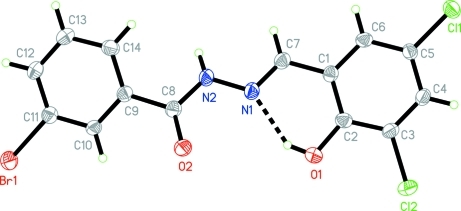
The molecular structure of the title compound, showing 30% probability displacement ellipsoids. The dashed line indicates a hydrogen bond.

**Fig. 2.**
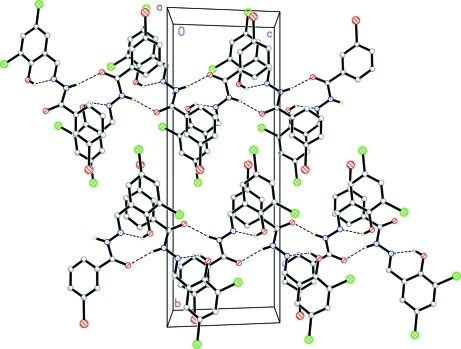
Molecular packing of the title compound, viewed along the a axis. Hydrogen bonds are shown as dashed lines.

## supplementary crystallographic information

### Comment

Schiff bases are readily synthesized by the reaction of aldehydes with primary amines (Akitsu & Einaga, 2006; Pradeep, 2005; Butcher *et al.*, 2005; Habibi *et al.*, 2007). We have reported a few Schiff bases and their complexes (Wei *et al.*, 2008; Zhu *et al.*, 2007; Wang *et al.*, 2006). In this paper, the crystal structure of a new Schiff base compound is reported.

The C═N bond length in the title molecule (Fig.1) is comparable with those observed in other Schiff bases (Yehye *et al.*, 2008; Odabaşoğlu *et al.*, 2007; Yathirajan *et al.*, 2007). All bond lengths are within normal ranges and are comparable to those observed in a related compound (Bao & Wei, 2008). The dihedral angle between C1—C6 and C9—C14 phenyl rings is 13.0 (2)°, indicating that the molecule is non-planar. An intramolecular O1—H1···N1 hydrogen bond is observed.

The crystal structure is stabilized by intermolecular N–H···O hydrogen bonds (Table 1), forming chains along the *c* axis (Fig. 2).

### Experimental

3,5-Dichloro-2-hydroxybenzaldehyde (1.0 mmol) and 3-bromobenzohydrazide (1.0 mmol) were dissolved in methanol (30 ml). The mixture was stirred at reflux for 10 min to give a clear colourless solution. After keeping this solution in air for 5 d, colourless needle-shaped crystals were formed.

### Refinement

Atom H2 was located in a difference Fourier map and refined isotropically, with the N–H distance restrained to 0.90 (1) Å. All other H atoms were positioned geometrically (C–H = 0.93 Å and O–H = 0.82 Å) and refined as riding, with *U*_iso_(H) values set at 1.2*U*_eq_(C) and 1.5*U*_eq_(O).

### Crystal data

**Table:**

C_14_H_9_BrCl_2_N_2_O_2_	*F*(000) = 768
*M**_r_* = 388.04	*D*_x_ = 1.743 Mg m^−^^3^
Monoclinic, *P*2_1_/*c*	Mo *K*α radiation, λ = 0.71073 Å
Hall symbol: -P 2ybc	Cell parameters from 1779 reflections
*a* = 8.272 (2) Å	θ = 2.5–24.9°
*b* = 22.366 (3) Å	µ = 3.15 mm^−^^1^
*c* = 8.237 (2) Å	*T* = 298 K
β = 104.014 (2)°	Cut from needle, colorless
*V* = 1478.6 (5) Å^3^	0.23 × 0.23 × 0.22 mm
*Z* = 4	

### Data collection

**Table:**

Bruker SMART 1000 CCD area-detector diffractometer	3224 independent reflections
Radiation source: fine-focus sealed tube	2144 reflections with *I* > 2σ(*I*)
graphite	*R*_int_ = 0.035
ω scans	θ_max_ = 27.0°, θ_min_ = 2.5°
Absorption correction: multi-scan (*SADABS*; Sheldrick, 1996)	*h* = −10→10
*T*_min_ = 0.495, *T*_max_ = 0.513	*k* = −25→28
8590 measured reflections	*l* = −9→10

### Refinement

**Table:**

Refinement on *F*^2^	Primary atom site location: structure-invariant direct methods
Least-squares matrix: full	Secondary atom site location: difference Fourier map
*R*[*F*^2^ > 2σ(*F*^2^)] = 0.036	Hydrogen site location: inferred from neighbouring sites
*wR*(*F*^2^) = 0.075	H atoms treated by a mixture of independent and constrained refinement
*S* = 0.98	*w* = 1/[σ^2^(*F*_o_^2^) + (0.0309*P*)^2^] where *P* = (*F*_o_^2^ + 2*F*_c_^2^)/3
3224 reflections	(Δ/σ)_max_ = 0.001
194 parameters	Δρ_max_ = 0.32 e Å^−^^3^
1 restraint	Δρ_min_ = −0.29 e Å^−^^3^

### Special details

**Table:**

Geometry. All e.s.d.'s (except the e.s.d. in the dihedral angle between two l.s. planes) are estimated using the full covariance matrix. The cell e.s.d.'s are taken into account individually in the estimation of e.s.d.'s in distances, angles and torsion angles; correlations between e.s.d.'s in cell parameters are only used when they are defined by crystal symmetry. An approximate (isotropic) treatment of cell e.s.d.'s is used for estimating e.s.d.'s involving l.s. planes.
Refinement. Refinement of *F*^2^ against ALL reflections. The weighted *R*-factor *wR* and goodness of fit *S* are based on *F*^2^, conventional *R*-factors *R* are based on *F*, with *F* set to zero for negative *F*^2^. The threshold expression of *F*^2^ > σ(*F*^2^) is used only for calculating *R*-factors(gt) *etc*. and is not relevant to the choice of reflections for refinement. *R*-factors based on *F*^2^ are statistically about twice as large as those based on *F*, and *R*- factors based on ALL data will be even larger.

### Fractional atomic coordinates and isotropic or equivalent isotropic displacement parameters (Å^2^)

**Table:**

	*x*	*y*	*z*	*U*_iso_*/*U*_eq_	
Br1	0.48270 (4)	0.491887 (13)	−0.27385 (4)	0.05628 (13)	
Cl1	1.05566 (10)	1.05415 (3)	0.26163 (9)	0.0498 (2)	
Cl2	1.25693 (10)	0.85535 (3)	0.61875 (9)	0.0575 (2)	
N1	0.7849 (3)	0.79256 (10)	0.0814 (3)	0.0410 (6)	
N2	0.6808 (3)	0.76119 (10)	−0.0450 (3)	0.0422 (6)	
O1	1.0195 (3)	0.79478 (8)	0.3556 (2)	0.0541 (5)	
H1	0.9462	0.7793	0.2827	0.081*	
O2	0.6767 (3)	0.68457 (8)	0.1345 (2)	0.0489 (5)	
C1	0.9228 (3)	0.88201 (12)	0.1849 (3)	0.0367 (6)	
C2	1.0236 (3)	0.85387 (12)	0.3269 (3)	0.0395 (7)	
C3	1.1329 (3)	0.88909 (12)	0.4431 (3)	0.0397 (7)	
C4	1.1435 (3)	0.94963 (12)	0.4228 (3)	0.0409 (7)	
H4	1.2183	0.9722	0.5017	0.049*	
C5	1.0430 (3)	0.97700 (11)	0.2850 (3)	0.0373 (7)	
C6	0.9340 (3)	0.94382 (12)	0.1660 (3)	0.0387 (7)	
H6	0.8676	0.9626	0.0726	0.046*	
C7	0.8091 (3)	0.84757 (12)	0.0558 (3)	0.0417 (7)	
H7	0.7548	0.8656	−0.0442	0.050*	
C8	0.6373 (3)	0.70501 (12)	−0.0078 (3)	0.0392 (7)	
C9	0.5343 (3)	0.67109 (12)	−0.1518 (3)	0.0373 (6)	
C10	0.5519 (3)	0.60935 (11)	−0.1486 (3)	0.0371 (6)	
H10	0.6248	0.5906	−0.0595	0.044*	
C11	0.4591 (3)	0.57631 (12)	−0.2802 (3)	0.0404 (7)	
C12	0.3488 (4)	0.60312 (13)	−0.4123 (3)	0.0468 (7)	
H12	0.2877	0.5801	−0.5000	0.056*	
C13	0.3297 (4)	0.66421 (13)	−0.4133 (4)	0.0500 (8)	
H13	0.2544	0.6826	−0.5013	0.060*	
C14	0.4222 (4)	0.69829 (13)	−0.2838 (3)	0.0444 (7)	
H14	0.4093	0.7396	−0.2852	0.053*	
H2	0.662 (4)	0.7733 (13)	−0.1513 (18)	0.080*	

### Atomic displacement parameters (Å^2^)

**Table:**

	*U*^11^	*U*^22^	*U*^33^	*U*^12^	*U*^13^	*U*^23^
Br1	0.0667 (2)	0.04067 (18)	0.0550 (2)	−0.00069 (16)	0.00200 (16)	−0.00918 (15)
Cl1	0.0677 (6)	0.0367 (4)	0.0459 (4)	−0.0006 (3)	0.0154 (4)	0.0005 (3)
Cl2	0.0656 (6)	0.0545 (5)	0.0430 (4)	0.0122 (4)	−0.0048 (4)	0.0070 (4)
N1	0.0484 (15)	0.0403 (14)	0.0325 (13)	−0.0066 (11)	0.0066 (11)	−0.0035 (11)
N2	0.0558 (16)	0.0401 (13)	0.0280 (12)	−0.0083 (12)	0.0053 (12)	−0.0038 (11)
O1	0.0718 (17)	0.0375 (11)	0.0463 (13)	−0.0037 (10)	0.0013 (11)	0.0071 (10)
O2	0.0714 (15)	0.0428 (11)	0.0283 (11)	−0.0042 (10)	0.0042 (10)	−0.0001 (9)
C1	0.0396 (16)	0.0403 (16)	0.0311 (14)	−0.0024 (13)	0.0103 (12)	−0.0019 (13)
C2	0.0462 (18)	0.0379 (16)	0.0361 (16)	0.0023 (13)	0.0130 (14)	0.0055 (13)
C3	0.0410 (17)	0.0448 (17)	0.0311 (15)	0.0044 (14)	0.0047 (13)	0.0022 (13)
C4	0.0429 (18)	0.0442 (17)	0.0335 (15)	−0.0037 (14)	0.0050 (13)	−0.0038 (13)
C5	0.0457 (19)	0.0329 (15)	0.0346 (15)	−0.0011 (12)	0.0119 (14)	−0.0007 (12)
C6	0.0467 (18)	0.0403 (16)	0.0295 (14)	0.0050 (14)	0.0100 (13)	0.0049 (13)
C7	0.0463 (19)	0.0455 (18)	0.0324 (15)	−0.0037 (14)	0.0076 (13)	−0.0004 (13)
C8	0.0462 (18)	0.0393 (16)	0.0317 (16)	0.0023 (13)	0.0085 (13)	−0.0041 (13)
C9	0.0430 (18)	0.0408 (16)	0.0280 (14)	−0.0024 (13)	0.0085 (13)	−0.0018 (13)
C10	0.0418 (17)	0.0370 (15)	0.0302 (15)	0.0013 (13)	0.0044 (12)	−0.0003 (12)
C11	0.0399 (18)	0.0410 (16)	0.0404 (17)	0.0012 (13)	0.0098 (14)	−0.0043 (14)
C12	0.0464 (19)	0.0520 (19)	0.0375 (17)	−0.0042 (15)	0.0016 (14)	−0.0060 (14)
C13	0.0449 (19)	0.056 (2)	0.0424 (18)	0.0007 (15)	−0.0023 (14)	0.0041 (15)
C14	0.0495 (19)	0.0385 (16)	0.0440 (17)	0.0040 (14)	0.0094 (15)	0.0053 (14)

### Geometric parameters (Å, °)

**Table:**

Br1—C11	1.898 (3)	C4—H4	0.93
Cl1—C5	1.742 (3)	C5—C6	1.377 (4)
Cl2—C3	1.731 (3)	C6—H6	0.93
N1—C7	1.272 (3)	C7—H7	0.93
N1—N2	1.372 (3)	C8—C9	1.490 (4)
N2—C8	1.362 (3)	C9—C14	1.387 (4)
N2—H2	0.893 (10)	C9—C10	1.388 (3)
O1—C2	1.345 (3)	C10—C11	1.381 (4)
O1—H1	0.82	C10—H10	0.93
O2—C8	1.227 (3)	C11—C12	1.377 (4)
C1—C6	1.397 (4)	C12—C13	1.375 (4)
C1—C2	1.410 (4)	C12—H12	0.93
C1—C7	1.458 (4)	C13—C14	1.381 (4)
C2—C3	1.391 (4)	C13—H13	0.93
C3—C4	1.370 (3)	C14—H14	0.93
C4—C5	1.377 (4)		
			
C7—N1—N2	117.7 (2)	N1—C7—H7	120.3
C8—N2—N1	116.9 (2)	C1—C7—H7	120.3
C8—N2—H2	120 (2)	O2—C8—N2	122.4 (2)
N1—N2—H2	121 (2)	O2—C8—C9	122.6 (2)
C2—O1—H1	109.5	N2—C8—C9	115.0 (2)
C6—C1—C2	119.5 (3)	C14—C9—C10	119.9 (3)
C6—C1—C7	119.3 (3)	C14—C9—C8	123.1 (2)
C2—C1—C7	121.1 (2)	C10—C9—C8	117.0 (2)
O1—C2—C3	118.4 (2)	C11—C10—C9	118.8 (3)
O1—C2—C1	123.3 (3)	C11—C10—H10	120.6
C3—C2—C1	118.3 (2)	C9—C10—H10	120.6
C4—C3—C2	121.7 (3)	C12—C11—C10	121.6 (3)
C4—C3—Cl2	119.4 (2)	C12—C11—Br1	119.9 (2)
C2—C3—Cl2	118.9 (2)	C10—C11—Br1	118.5 (2)
C3—C4—C5	119.8 (3)	C13—C12—C11	119.4 (3)
C3—C4—H4	120.1	C13—C12—H12	120.3
C5—C4—H4	120.1	C11—C12—H12	120.3
C4—C5—C6	120.5 (3)	C12—C13—C14	120.2 (3)
C4—C5—Cl1	119.4 (2)	C12—C13—H13	119.9
C6—C5—Cl1	120.1 (2)	C14—C13—H13	119.9
C5—C6—C1	120.2 (3)	C13—C14—C9	120.2 (3)
C5—C6—H6	119.9	C13—C14—H14	119.9
C1—C6—H6	119.9	C9—C14—H14	119.9
N1—C7—C1	119.4 (3)		

### Hydrogen-bond geometry (Å, °)

**Table:**

*D*—H···*A*	*D*—H	H···*A*	*D*···*A*	*D*—H···*A*
O1—H1···N1	0.82	1.88	2.598 (3)	145
N2—H2···O2^i^	0.89 (1)	2.03 (1)	2.898 (3)	165 (3)

Symmetry codes: (i) *x*, −*y*+3/2, *z*−1/2.
